# The role of dietary fatty acid intake in inflammatory gene expression: a critical review: O papel da ingestão dos ácidos graxos da dieta na expressão de genes inflamatórios: uma revisão crítica

**DOI:** 10.1590/1516-3180.2016.008607072016

**Published:** 2017-01-05

**Authors:** Daniela Mayumi Rocha, Josefina Bressan, Helen Hermana Hermsdorff

**Affiliations:** I RD, MSc. Department of Nutrition and Health, Universidade Federal de Viçosa (UFV), Viçosa (MG), Brazil.; II RD, MSc, PhD. Titular Professor, Department of Nutrition and Health, Universidade Federal de Viçosa (UFV), Viçosa (MG), Brazil.; III RD, MSc, PhD. Assistant Professor, Department of Nutrition and Health, Universidade Federal de Viçosa (UFV), Viçosa (MG), Brazil.

**Keywords:** Dietary fats, Fatty acids, Gene expression, Inflammation, Dietary fats, unsaturated

## Abstract

**CONTEXT AND OBJECTIVE:**

Diet is an important modifiable factor involved in obesity-induced inflammation. We reviewed clinical trials that assessed the effect of consumption of different fatty acids on the expression of inflammation-related genes, such as cytokines, adipokines, chemokines and transcription factors.

**DESIGN AND SETTING:**

Narrative review study conducted at a research center.

**METHODS:**

This was a review on the effect of fat intake on inflammatory gene expression in humans.

**RESULTS:**

Consumption of saturated fatty acids (SFAs) was related to postprandial upregulation of genes associated with pro-inflammatory pathways in peripheral blood mononuclear cells (PBMCs), in comparison with monounsaturated fatty acid (MUFA) or polyunsaturated fatty acid (PUFA) intake. In addition, acute intake of a high-SFA meal also induced a postprandial pro-inflammatory response for several inflammatory genes in subcutaneous adipose tissue. Both high-MUFA and high-PUFA diets showed anti-inflammatory profiles, or at least a less pronounced pro-inflammatory response than did SFA consumption. However, the results concerning the best substitute for SFAs were divergent because of the large variability in doses of MUFA (20% to 72% of energy intake) and n3 PUFA (0.4 g to 23.7% of energy intake) used in interventions.

**CONCLUSIONS:**

The lipid profile of the diet can modulate the genes relating to postprandial and long-term inflammation in PBMCs and adipose tissue. Identifying the optimal fat profile for inflammatory control may be a promising approach for treating chronic diseases such as obesity.

## RESUMO

**CONTEXTO E OBJETIVO:**

A dieta é um importante fator modificável envolvido na inflamação induzida pela obesidade. Nós revisamos ensaios clínicos que avaliaram o efeito do consumo de diferentes ácidos graxos sobre a expressão de genes relacionados com a inflamação, tais como citocinas, adipocitocinas, quimiocinas e fatores de transcrição.

**TIPO DE ESTUDO E LOCAL:**

Estudo de revisão narrativa realizado em um centro de pesquisa.

**MÉTODOS:**

Revisão do efeito da ingestão de gordura sobre a expressão de genes envolvidos com inflamação em seres humanos.

**RESULTADOS:**

O consumo do ácido graxo saturado (AGS) foi relacionado com a regulação favorável pós-prandial de genes associados com vias pró-inflamatórias nas células mononucleares de sangue periférico (CMSP), em comparação com a ingestão do ácido graxo monoinsaturado (AGMI) ou do ácido graxo poli-insaturado (AGPI). Além disso, o consumo agudo de uma dieta com alto conteúdo de AGS também induziu uma resposta pró-inflamatória pós-prandial para vários genes da inflamação no tecido adiposo subcutâneo. Ambas as dietas com alto conteúdo de AGMI e AGPI apresentaram perfil anti-inflamatório ou, pelo menos, menor resposta pró-inflamatória em relação ao consumo de AGS. Contudo, os resultados são controversos acerca do melhor substituto para o AGS, devido à grande variabilidade na dose de AGMI (20% a 72% da ingestão energética) e AGPI n3 (0,4 g para 23,7% da ingestão energética) utilizados nos estudos de intervenção.

**CONCLUSÕES:**

O perfil lipídico da dieta pode modular os genes relacionados com inflamação pós-prandial e a longo prazo em CMSP e no tecido adiposo. Identificar o perfil lipídico ideal no controle inflamatório pode ser uma abordagem promissora para o tratamento de doenças crônicas como a obesidade.

## INTRODUCTION

Inflammation is a physiological response triggered by infection and injury that has the purposes of eliminating irritating agents and accelerating tissue regeneration.^1^^,^^2^ In this process, several inflammatory mediators are released, including cell adhesion molecules, cytokines, chemokines and other inflammatory agents (e.g. nitrogen and reactive oxygen species).^3^ In order to maintain the homeostatic balance, a controlled inflammatory response is required. On the other hand, excessive or inappropriate inflammation leads to a pathological inflammatory status.^1^ Increasingly, there is evidence to suggest that a deregulated inflammatory response plays a pivotal role in the onset and progression of atherosclerosis.^4^

Moreover, excessive adiposity and adiposity-related metabolic diseases (metabolic syndrome, diabetes and atherosclerosis) are attributed to a chronic state of low-grade inflammation. Therefore, diet-induced weight loss is an important factor for reducing pro-inflammatory markers.^5^^,^^6^^,^^7^ In fact, besides lipid storage, fat cells are capable of producing and secreting chemoattractants such as monocyte chemotactic protein-1 (MCP-1) and pro-inflammatory mediators such as interleukins (IL), for instance IL-1β, IL-6 and tumor necrosis factor (TNF)-α, during adipose tissue expansion, thereby resulting in inflammatory and metabolic deregulation.^8^

Many environmental factors can contribute towards obesity and thus interfere with inflammatory expression, including diet.^9^ Nutritional interventions can modulate inflammation, as demonstrated in studies based on a hypocaloric diet or on high consumption of fruits and vegetables. Both interventions have been shown to reduce the expression and synthesis of pro-inflammatory cytokines (IL-6 and TNF-α) and decrease other inflammatory markers such as C-reactive protein (CRP).^10^^,^^11^^,^^12^ In addition, previous studies have confirmed that high-fat meals reduce leptin concentrations and increase the activation of inflammatory markers such as IL-6 during the postprandial phase.^13^^,^^14^

In fact, fatty acids can directly or indirectly modify immune and inflammatory responses. Current evidence suggests that a family of receptors involved in innate immunity, known as Toll-like receptors (TLRs), is connected with the inflammatory response relating to saturated fatty acid (SFA) intake. In this regard, it has been proposed that SFAs are nonmicrobial TLR agonists that promote inflammatory activation.^15^ Studies have shown that the SFA lauric acid stimulates pro-inflammatory expression by TLR2 and TLR4, thereby mediating nuclear factor kappa B (NF-κB) and cyclooxygenase-2 activation and expression. In contrast, consumption of fish oil rich in n3 polyunsaturated fatty acid (PUFA) inhibits the TLR4-induced signaling pathways and target gene expression.^16^^,^^17^ Moreover, SFA intake is known to cause lipemia that is more pronounced than the lipemia due to monounsaturated fatty acids (MUFA) and PUFA, which can lead to a higher pro-inflammatory state associated with SFA consumption.^18^ Additionally, SFA palmitate and stearate acids can trigger IL-1β secretion through mechanisms involving NLRP3 (NOD-like receptor family, pyrin domain containing 3) inflammasome activation.^19^

Recently, it was proposed that GPR120 (G protein-coupled receptor 120) mediates the anti-inflammatory effects of n3 PUFA.^20^^,^^21^ Dietary n3 PUFA has been correlated with inhibition of TLR-induced signaling pathways and target gene expression, probably through disruption of translocation of TLR4 into a lipid raft.^16^^,^^17^ In combination, these mechanisms can potentially inhibit the signaling pathways that lead to NF-κB activation, thus resulting in downregulation of pro-inflammatory responses through n3 PUFA intake.

## OBJECTIVE

Given the above, we aimed to summarize and discuss recent evidence about the effect of consumption of different fatty acids in humans, on inflammation-related gene expression, as evaluated through clinical studies.

## METHODS

This was a narrative review of the English-language literature on the effects of fat intake on inflammatory gene expression in humans. It evaluated studies indexed in the Cochrane Library, LILACS and PubMed databases between the time of database inception and March 2016 (Table 1). We included original studies that reported on clinical trials on men or women (not pregnant, not in lactation and not in the postmenopausal period) who were not athletes, not undergoing hormonal treatment, not dependent on alcohol or drugs and not suffering from chronic illnesses (such as hepatic, renal, thyroid or cardiac dysfunction) or acute inflammatory processes. Since the objective was to evaluate the effect of fatty acid consumption among humans, only clinical trials were included, given that these are considered to be the mainstay design for causal inferences.

**Table 1:** Database search results

Database	Search	Filters		Results
Cochrane Library	("gene expression" OR "RNA" OR "mRNA" OR "gene") AND (("saturated fatty acid" OR "saturated fatty acids" OR "SFA" OR "SFAs") OR ("monounsaturated fatty acid" OR "monounsaturated fatty acids" OR "MUFA" OR "MUFAs") OR ("polyunsaturated fatty acid" OR "polyunsaturated fatty acids" OR "PUFA" OR "PUFAs")) AND ("inflammation" OR "inflammatory" OR "proinflammatory")	Title, abstract, keywords in trials	48 articles	2 animal/*in vitro* studies 3 poster session abstracts 43 clinical trials
LILACS	("gene expression" OR "RNA" OR "mRNA" OR "gene") AND (("saturated fatty acid" OR "saturated fatty acids" OR "SFA" OR "SFAs") OR ("monounsaturated fatty acid" OR "monounsaturated fatty acids" OR "MUFA" OR "MUFAs") OR ("polyunsaturated fatty acid" OR "polyunsaturated fatty acids" OR "PUFA" OR "PUFAs")) AND ("inflammation" OR "inflammatory" OR "proinflammatory")	No filter	2 articles	2 reviews
PubMed	("gene expression" OR "RNA" OR "mRNA" OR "gene") AND (("saturated fatty acid" OR "saturated fatty acids" OR "SFA" OR "SFAs") OR ("monounsaturated fatty acid" OR "monounsaturated fatty acids" OR "MUFA" OR "MUFAs") OR ("polyunsaturated fatty acid" OR "polyunsaturated fatty acids" OR "PUFA" OR "PUFAs")) AND ("inflammation" OR "inflammatory" OR "proinflammatory")	Title/abstract in clinical trials on humans	44 articles	4 animal/*in vitro* studies 40 clinical trials

All the papers were checked according to their titles and abstracts (screening). Full papers were obtained from journals available on the CAPES Foundation (Ministry of Education, Brazil) website. Unavailable articles were requested from their authors. Articles presenting potentially relevant studies were read and analyzed to assess the inclusion criteria.

**Figure 1:**
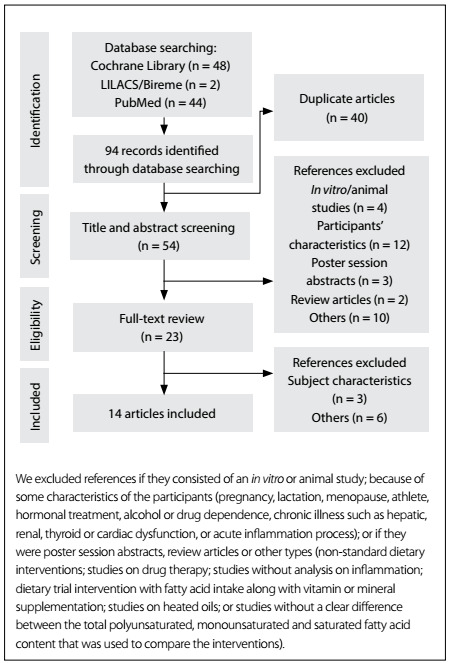
Literature search process.

We excluded articles that consisted of *in vitro* or animal studies, articles in which the participants' characteristics did not match those mentioned above, poster session abstracts, review articles and other types of publications (non-standard dietary interventions; studies on drug therapy; studies without any analysis on inflammation; dietary trial interventions on fatty acid intake along with vitamin or mineral supplementation; studies on heated oils; or studies without any clear differentiation between the total polyunsaturated, monounsaturated and saturated fatty acid content used to compare the interventions). The flowchart for the study selection process is described in Figure 1. Other papers were used for contextualization and discussion.

## RESULTS

We identified 14 studies that investigated the effect of fatty acid intake on inflammatory gene expression (Table 2). Six of these studies had a postprandial design in which an acute inflammatory response to a high-fat meal consumed on a single day was evaluated^22^^,^^23^ or consisted of a postprandial fat challenge, reflecting fat composition similar to that of a dietary intervention conducted for at least four weeks afterwards.^24^^,^^25^^,^^26^^,^^27^Postprandial is a term that was introduced in 1997 and refers to "the time frame after a meal or food intake".^28^ Seven studies assessed the inflammatory response after long-term consumption (minimum of 8 weeks).^29^^,^^30^^,^^31^^,^^32^^,^^33^^,^^34^^,^^35^Lastly, one study determined both the postprandial and the long-term response to the same dietary intervention.^36^ In order to assist comparisons between the studies, dietary interventions were compared according to fat content source (SFA, MUFA or PUFA) and its respective proportion of total energy intake (E%).

**Table 2:** Summary of the studies selected

Year, authors	Subjects	Study design	Dietary intervention	Duration	Postprandial intervention	Inflammatory gene markers
2009, Bouwens et al.^29^	n = 111 M/F: 66/45 Age: 66-80 years	Clinical trial, randomized, double-blind, controlled, parallel	3 groups (N/A E% from fat): - High n3 PUFA (EPA/DHA): 1.8 g/day - Control (sunflower oil): 4.0 g/day	26 weeks	-	RNA microarray analysis (encoding 17,699 genes) in PBMCs, measured in fasting period at baseline and after the intervention period.
2009, van Dijk et al.^33^	n = 20 M/F: 10/10 Mean age: 45-60 years	Clinical trial, parallel	2 groups (N/A E% from fat): - SFA: 19 E% - MUFA: 20 E%	8 weeks	-	RNA microarray analysis (encoding 17,699 genes) in SAT, measured in fasting period at baseline and after the intervention period.
2009, Jiménez-Gómez et al.^24^	n = 20 M/F: 20/0 Mean age: N/A	Clinical trial, randomized, crossover, postprandial	3 groups: High fat (38 E% from fat): - SFA: 22 E% - MUFA: 24 E% Low fat (< 30% E% from fat): - n3 PUFA (ALA): 2 E%	4 weeks	Breakfast (60 E% from fat): - SFA: 35 E% - MUFA: 36 E% - n3 PUFA (ALA): 4 E%	TNF-α, IL-6 and MCP-1 in PBMCs, measured in fasting period and 3, 6 and 9 h after the breakfast.
2011, Meneses et al.^25^	n = 39 M/F: 14/25 Mean age: ~ 57 years	Clinical trial, randomized, controlled, parallel, postprandial	4 groups: High fat (38 E% from fat): - SFA: 16 E% - MUFA: 20 E% Low fat (28 E% from fat): - LFHCC n3: supplement with 1.24 g/day of n3 PUFA - LFHCC: 1.2 g/day supplement of control (sunflower oil)	12 weeks	Breakfast (65 E% from fat): - SFA: 38 E% - MUFA: 43 E% - LFHCC n3: supplement with 1.24 g of n3 PUFA - LFHCC: supplement with placebo capsules	p65, IκBα, IκBβ2, IL-6, MCP-1 and IL-1β in SAT, measured in fasting period and 4 h after the breakfast.
2011, Pietraszek et al.^23^	n = 34 M/F: 11/23 Mean age: ~ 50 years	Clinical trial, randomized, crossover, postprandial	Standard diet: 24 E% from fat	1 day	Breakfast (87 E% from fat): - SFA: 79 E%^§^ - MUFA: 72 E%	ADIPOR1, ADIPOR2, MCP-1, IL-1β, IL-6, IL-6R, CD16A, LEP, LEPR, RBP4, TLR4, TNF-α AND TNFRSF1A in muscle and SAT, measured in fasting period and 3 h 30 min after the breakfast. Also, ADIPQ was determined in SAT.
2011, Rudkowska et al.^34^	n = 16 M/F: 7/9 Mean age: 57 years	Clinical trial, randomized, crossover	2 groups: - n3 PUFA: 1.8 g/day - n3 PUFA FP: 1.8 g/day + fish protein	8 weeks	-	RNA microarray analysis (encoding 37,804 genes) in PBMCs, measured at baseline and after the intervention period.
2012, Camargo et al.^26^	n = 20 M/F: 10/10 Mean age: 67 years	Clinical trial, randomized, crossover, postprandial	3 groups: High fat (38 E% from fat): - SFA: 22 E% - MUFA: 24 E% Low fat (< 30% E% from fat): - n3 PUFA (ALA): 2 E%	4 weeks	Breakfast (60 E% from fat): - SFA: 35 E% - MUFA: 36 E% - n3 PUFA (ALA): 4 E%	p65, IκBα, MCP-1, MIF-1, MMP-9 and IL-6 in PBMCs, measured in fasting period and 1, 2 and 4 h after the breakfast.
2012, Cruz-Teno et al.^27^	n = 75 M/F: 28/47 Mean age: ~ 56 years	Clinical trial, randomized, controlled, parallel, postprandial	4 groups: High fat (38 E% from fat): - SFA: 16 E% - MUFA: 20 E% Low fat (28 E% from fat): - LFHCC n3: supplement with 1.24 g/day of n3 PUFA - LFHCC: 1.0 g/day supplement of control (sunflower oil)	12 weeks	Breakfast (65 E% from fat): - SFA: 38 E% - MUFA: 43 E% - LFHCC n3: supplement with 1.24 g of n3 PUFA - LFHCC: supplement with placebo capsules	TNF-α, IL-6, IκB-α, p65, MCP-1, MIF, MMP-9 in PBMCs, measured in fasting period and 2 and 4 h after the breakfast.
2012, van Dijk et al.^30^	n = 49 M/F: 22/27 Mean age: ~ 55 years	Clinical trial, parallel	3 groups (37-40 E% from fat): - SFA: 19 E% - MUFA: 20% E% - MED^*^: 21 E% from MUFA	8 weeks	-	RNA microarray analysis (encoding 17,699 genes) in PBMCs, measured in fasting period at baseline and after the intervention period.
2012, van Dijk et al.^22^	n = 42 M/F: 42/0 Age: 50-70 years	Clinical trial, randomized, double-blind, crossover, postprandial	Low-fat meal: N/A	1 day	Shake (87 E% from fat): - SFA: 46.5 E% - MUFA: 72 E% - n3 PUFA (EPA/DHA): 23.7 E%	IL-1β, IL-8, MCP-1, NFkB1 and TNF-α in PBMCs, measured in fasting period and 2 and 4 h after the breakfast.
2012, Schmidt et al.^36^	n = 40 M/F: 40/0 Mean age: ~ 40 years	Clinical trial, randomized, double-blind, controlled, parallel, postprandial	2 groups: - n3 PUFA (EPA/DHA): 2.7 g/day - Control (n6 PUFA): 3.05 g/day (LA)	12 weeks	Consumption of capsules of the dietary intervention.	RNA microarray analyses from whole blood at baseline, after 1 and 12 weeks of supplementation (long-term effect). Also 4 h after intake of capsules (postprandial response).
2012, Itariu et al.^31^	n = 55 M/F: 46/9 Mean age: ~ 38 years	Clinical trial, randomized, controlled, parallel	2 groups (30 E% from fat): - n3 PUFA (EPA/DHA): 3.36 g/day - Control (butter): 5 g/day	8 weeks	-	MCP-1, MIP-1α, IL-6, ADIPOQ, HIF1A and TGF-β1, CD68, CD163, MRC1 and CD40 in SAT and VAT, after the intervention period.
2013, Kratz et al.^32^	n = 24 M/F: 8/16 Mean age: ~ 39 years	Clinical trial, randomized, single-blind, controlled, parallel	2 groups (~34 E% from fat): - n3 PUFA: 3.5 E% - Control: 0.5 E% from n3 PUFA	14 weeks	-	TNF-α, IL-6, ICAM-1, CD14, CD206, CD284, MCP-1 and SAA1 in SAT, collected at baseline and after the intervention period.
2013, Labonté et al.^35^	n = 12 M/F: 12/0 Mean age: 54 years	Clinical trial, randomized, double-blind, controlled, crossover	2 groups: - n3 PUFA (EPA/DHA): 3.0 g/day - Control: blend of corn and soybean oil	8 weeks	-	IL-6, IL-18, TNF-α in duodenal samples, collected in fasting period at the end of each intervention.

n = number of subjects; M/F = male/female; N/A = not available; SFA = saturated fatty acids; MUFA = monounsaturated fatty acids; n3 PUFA = n3 polyunsaturated fatty acids; LFHCC n3 = high complex carbohydrate supplemented with n3 PUFA; LFHCC = high complex carbohydrate supplemented with placebo; ALA = α-linolenic acid; LA = linoleic acid; E% = % of energy intake; PBMC = peripheral blood mononuclear cells; SAT = subcutaneous adipose tissue; VAT = visceral adipose tissue; ADIPOR = adiponectin receptor; ADIPQ = adiponectin gene; CD14, CD163, CD16A, CD206, CD284, CD40, CD68 = macrophage markers; HIF1α = hypoxia-induced factor 1α; ICAM-1 = intercellular adhesion molecule-1; IL = interleukin; IκB = inhibitor of NF-κB; LEP = leptin; LEPR = leptin receptor; MCP-1 = monocyte chemoattractant protein-1; MIF = macrophage migration inhibitory factor; MIP-1 = macrophage inflammatory protein 1; MMP-9 = matrix metalloproteinase 9; MRC1 = mannose receptor C type 1; NFkB1 = nuclear factor kappa-B subunit 1; p65 = nuclear p65 protein; RBP4 = retinol binding protein 4; SAA1 = serum amyloid A1; TGF-β1 = transforming growth factor β1; TLR4 = Toll-like receptor 4; TNF-α = tumor necrosis factor alpha; TNFRSF1A = tumor necrosis factor receptor superfamily member 1A.

*Mediterranean (MED) diet components high in MUFA from extra-virgin olive oil and containing additional MED components (i.e. fatty fish, unrefined grain products, nuts, legumes and red wine).

^§^The SFA meal was high in coconut oil with 49 E% from medium-chain SFA (predominantly lauric acid) and 30 E% from long-chain SFA (predominantly myristic acid).

Inflammatory genes were analyzed in duodenal tissue, peripheral blood mononuclear cells (PBMCs), subcutaneous or visceral adipose tissue, skeletal muscle and whole blood, mostly using the polymerase chain reaction (PCR), or using a microarray analysis methodology. The main inflammatory genes screened were those that promote expression of adipokines, chemokines, cytokines and transcription factors. Hence, before discussing the role of fatty acid intake in inflammatory gene expression, we firstly contextualize the main markers that have been found in various studies.

### Adipokines

Adipose tissue is an active organ involved not only in energy storage control, but also in regulation of complex metabolic and endocrine functions. In this context, adipose tissue releases cytokines and other bioactive mediators. Adiponectin and leptin are known as true adipokines, and are the major adipocyte proteins produced mainly by adipose tissue.^37^^,^^38^ In particular, adiponectin is an anti-inflammatory cytokine that might be able to induce production of other anti-inflammatory cytokines such as IL-10 and IL-1 receptor antagonist (IL-1RA). At the same time, it may suppress pro-inflammatory cytokine production of interferon (IFN)-γ^37^ and may also have a negative correlation with CRP, the systemic inflammatory marker.^39^ Moreover, adiponectin can exhibit atheroprotective effects, through attenuating chronic inflammation in vascular walls.^40^ On the other hand, leptin correlates directly with body fat mass and adipocyte size, and has a role as a pro-inflammatory cytokine. Leptin stimulates production of several inflammatory mediators such as IL-1, IL-6, IL-12 and TNF.^37^ In addition, leptin has been correlated with several obesity-associated diseases such as cardiovascular diseases and diabetes.^41^

### Chemokines

Chemokines form a family of small proteins that are secreted in response to signals such as pro-inflammatory cytokines. They play an important role in selectively inducing chemotaxis and chemokinesis of leukocytes.^42^ MCP-1, also referred to as chemokine ligand 2 (CCL2), is a potent chemoattractant of monocytes and macrophages to inflammation areas, expressed mainly by inflammatory cells and endothelial cells.^43^^,^^44^ Macrophage inflammatory protein 1 (MIP-1), or chemokine ligand 3 (CCL3), is another potent chemoattractant of immune cells, particularly macrophages, to inflammatory sites.^43^

Leukocyte extravasation into tissues requires not only chemokines but also matrix-degrading enzymes, in particular matrix metalloproteinases (MMPs). MMP-9, for instance, performs an important role in immune cell functioning and in pathophysiological conditions that involve inflammatory processes. In addition, MMP-9 levels increase in cases of cardiovascular diseases, including hypertension, atherosclerosis and myocardial infarction.^45^^,^^46^

### Cytokines

Cytokines are proteins that act as intracellular mediators. They play an important role in cell communication and regulation of the immune system. Unlike classical hormones, they are produced by different tissues and cell types rather than by specialized glands. They bind to their cognate receptors on target cells and activate or inhibit cellular functions in a paracrine or autocrine manner.^47^^,^^48^

IL-1 was the first interleukin to be identified. It is produced by numerous innate immune cells including monocytes, macrophages and dendritic cells.^49^ IL-1 is a key pro-inflammatory mediator involved in hosting responses to pathogens and inflammation. Its synthesis is induced by other inflammatory cytokines such as TNF-α, IFN-γ and IL-2.^50^ IL-1 also induces production of pro-inflammatory cytokines, including IL-6.^51^ Growing evidence links this cytokine to chronic diseases such as type 2 diabetes and obesity. Furthermore, IL-1 is also related to atherosclerosis development.^52^

IL-6 is known as an inflammatory cytokine secreted mainly (10-35%) by adipose tissue, and also by skeletal muscle and liver.^53^ It is an acute-response mediator and consequently increases the plasma concentrations of acute-phase proteins, such as CRP and serum amyloid A.^54^ Greater IL-6 concentration is correlated with elevated cardiovascular risk. This can be explained by its correlations with increased adiposity, expressed in terms of body mass index, waist circumference, visceral fat, total body fat and increased risk of insulin resistance.^2^^,^^6^^,^^55^ Furthermore, high consumption of fruit and vegetables followed by good adherence to a calorie-restricted diet based on a Mediterranean dietary pattern can reduce expression and synthesis of this inflammatory marker, as described previously.^10^^,^^12^

IL-8 is a pro-inflammatory cytokine produced mainly by monocytes and macrophages. It is responsible for bringing immune cells to sites of inflammation and retaining them there. In addition, IL-8 promotes activation of monocytes and neutrophils. It has been shown to have a potential role in cardiovascular diseases, in particular atherosclerosis.^56^

TNF-α, also known as cachectin, is a strong pro-inflammatory cytokine produced mainly by monocytes and macrophages, via activation of MAPK (mitogen-activated protein kinase) and NF-κB signaling pathways. This process results in release of inflammatory genes and other inflammatory cytokines such as IL-1β and IL-6.^57^^,^^58^ TNF-α has also been implicated in increased cardiovascular risk, and it is central to the pathophysiology of cancer and chronic inflammatory conditions, including inflammatory bowel diseases, rheumatoid arthritis and psoriasis.^57^^,^^59^^,^^60^ Similarly to IL-6, a reduced-calorie diet and consumption of fruits and vegetables can reduce synthesis and expression of TNF-α.^10^^,^^12^

### Transcription factors

Nuclear p65 protein is a subunit of NF-κB transcription complex, which plays a crucial role in inflammatory and immune responses. NF-κB is a homo or heterodimer composed of Rel proteins: p65 (RelA), p50 (NFKB1), p52 (NFKB2), c-Rel and RelB.^61^ NF-κB is classically activated by pro-inflammatory cytokines such as TNF-α and alternatively by cytokines such as lymphotoxin β. An atypical pathway triggered by DNA damage^62^ may also do the activation. Altered NF-kB activation has been demonstrated in tumor development and chronic inflammatory diseases.^62^ Interestingly, high intake of fruits and vegetables is inversely associated with mRNA p65 expression in PBMCs of healthy adults.^10^

NF-κB signaling is controlled through NF-κB inhibitors (IκB). This is a family of proteins that can bind NF-κB dimers in the cytoplasm and nucleus, thereby inhibiting the NF-κB transcriptional response.^63^ Certain stimuli result in phosphorylation, and subsequent proteasome-mediated degradation of IkB proteins allows the unbound NF-κB dimers to translocate to the nucleus, thereby regulating the expression of target genes.^62^

### Fatty acid intake in inflammatory gene expression 

According to the studies reviewed here, the postprandial period resulted in a pro-inflammatory response regarding PBMC gene expression, linked to SFA consumption. In healthy subjects, SFA intake (35 E%) from animal sources resulted in an increased postprandial pro-inflammatory response in PBMCs for TNF-α expression, in comparison with MUFA (36 E%) and n3 PUFA (4 E%) breakfasts consisting mainly of extra-virgin olive oil and fats of vegetable origin (walnuts), respectively. In addition, mRNA IL-6 postprandial expression was higher after the high-SFA meal than after the n3 PUFA meal. However, the increased gene expression did not change the concentration of the inflammatory cytokines.^24^ Thus, the length of postprandial assessment time may have been sufficient to detect differences in expression but not in translation of cytokines in PBMCs. These differences between inflammatory gene response and synthesis/secretion of inflammatory markers may have been due to transcriptional and translational process that do not occur simultaneously.^64^ Moreover, several translational and post-translational regulatory mechanisms, including miRNA, may be involved and thus may affect the production and release of cytokines,^65^ which may also not occur concurrently in the cell and extracellular tissues. In addition, discrepancies can be found between cytokine concentrations and their mRNA expression, probably due to potential confounding factors such as gender, physical activity, smoking and body mass index.^5^ However, PBMCs are widely used for determining inflammatory gene expression, given the fact that they are accessible cells. Furthermore, their use is cost-effective and they provide a less invasive alternative to biopsy measurements.^66^

Elderly subjects exhibited a pro-inflammatory response in PBMCs relating to high SFA consumption (35 E%), with higher postprandial inflammatory expression of p65 and MCP-1 genes, compared with MUFA (36 E%) mainly from virgin olive oil. They also presented higher mRNA p65, in comparison with an intervention comprising PUFA (4 E%) from plant origin (walnuts). Additionally, SFA showed downregulated expression of anti-inflammatory genes (IκBα), compared with MUFA, and increased plasma concentration of MCP-1 pro-inflammatory cytokines.^26^

In metabolic syndrome patients, SFA consumption (38 E%) was associated with upregulation of pro-inflammatory genes (MMP-9 and TNF-α) and downregulation of anti-inflammatory genes (IκBα) in a postprandial state, compared with MUFA (43 E%), in PBMCs. Moreover, higher MCP-1 plasma concentration was observed in SFA consumption, compared with MUFA and n3 PUFA (1.24 g). Regardless of the type of ingested fat (SFA, MUFA or n3 PUFA), the postprandial state was associated with increased expression of IL-6, MMP-9 and TNF-α pro-inflammatory genes, as well as higher IL-6 plasma concentrations,^27^ thus suggesting that a greater inflammatory response would be expected in these subjects. In fact, non-dietary factors, such as obesity and type 2 diabetes, can increase the extent of fatty acid postprandial inflammatory response.^67^ However, the source of fats was not mentioned in the study and it is known that dietary fat sources differ in more aspects than only their fatty acid profiles.

In this regard, olive oil is well known for its potential health-promoting properties, which are due to the presence of high levels of MUFA and other valuable minor components such as phenolics, phytosterols, tocopherols, carotenoids, chlorophyll and squalene.^68^ These natural compounds with antioxidant and other potentially important types of bioactivity have a beneficial impact on inflammatory markers.^67^ Thus, they represent an important confounding factor in assessing the effect of dietary fat intake on the inflammatory response.

Controversially, high-SFA acute intake (46.5 E%) mainly from plant origin (palm oil) was associated with reduced postprandial inflammatory response regarding PBMC gene expression (MCP-1 and IL-8), compared with MUFA (72 E%) from high-oleic acid sunflower oil and n3 PUFA (23.7 E%) from fish oil interventions.^22^ Palm oil use is subject to debate with regard to potential unhealthy effects, because of its high palmitic acid content. An increased inflammatory response (IL-6) relating to a palmitic oil-enriched diet in mice and a similar effect from palmitic acid *in vitro* was shown in one study. However, apart from SFAs, which are mostly from palmitic acid, this plant oil contains oleic and linoleic acids, which are MUFA and PUFA, respectively.^69^ Unlike in other studies, a much higher amount of MUFA was used, in comparison with the SFA intervention. In addition, n3 PUFA intake was greater than in other studies.

However, in a long-term dietary trial on the inflammatory response in PBMCs, gene expression remained unchanged after eight weeks of intervention with SFAs (19 E%), among abdominally obese patients.^30^ This result may be related to the lower amount of fat provided, in comparison with other interventions. Moreover, presence of the obese phenotype was correlated with a previous abnormal inflammatory profile.^30^

On the other hand, in subcutaneous adipose tissue among abdominally overweight subjects, investigation of a long-term SFA diet (19 E%) regarding the inflammatory response showed that upregulation of genes mainly relating to immune and inflammatory pathways occurred. At the same time, downregulation of anti-inflammatory genes and reduction of plasma adiponectin concentration were also observed.^33^ Among healthy subjects, an acute dietary intervention that was high in medium-chain SFAs (79 E%), i.e. rich in coconut oil, induced a postprandial pro-inflammatory response relating to several inflammatory genes in subcutaneous adipose tissue (CD16a, IL-1β, IL-6, IL-6R and TNF-α) and muscle tissue (MCP-1, IL-6R, CD16a, LEP, TLR4 and TNF-α). Additionally, plasma IL-6 concentration increased in response to medium-chain SFA consumption.^23^ In this regard, SFA appears to be able to modulate gene expression in important sources of inflammatory markers, such as PBMCs and adipose tissue.

Regarding the effect of MUFA consumption on inflammatory gene expression in subcutaneous adipose tissue, acute MUFA intake (72 E%) containing macadamia nut oil induced a postprandial anti-inflammatory response (ADIPOQ) in healthy subjects. However, it also increased the pro-inflammatory gene expression (TNFRSF1A), but in a less pronounced manner than did SFA (79 E%) derived from coconut oil intake.^23^ Moreover, a long-term MUFA (20 E%) dietary intervention, mainly in the form of refined olive oil, among abdominally obese subjects for eight weeks, also resulted in downregulation or unchanged expression of pro-inflammatory genes in subcutaneous adipose tissue, compared with a SFA diet (19%).^33^ These results indicate that MUFA can also exert a pro-inflammatory response, but only weakly, compared with SFA consumption. In fact, unlike SFAs, unsaturated fatty acids such as oleate acid were unable to activate the NLRP3 inflammasome and thereby stimulate IL-1β production.^19^ However, other mechanisms may be involved in the inflammatory response mediated by MUFA intake, which can elicit a pro or anti-inflammatory response.

Among subjects at higher risk of type 2 diabetes, an acute postprandial intervention of MUFA breakfast (72 E%) containing macadamia nut oil showed that several inflammatory genes were upregulated in subcutaneous adipose tissue (MCP-1, IL-1β, IL-6, IL-6R, TNF-α and TNFRSF1A). However, healthy subjects showed upregulation of proinflammatory genes (TNFRSF1A) but also of anti-inflammatory ones such as ADIPOQ.^23^ Metabolic syndrome patients also showed an increased postprandial response of inflammatory genes (p65, MCP-1, IL-6 and IL-1β) and anti-inflammatory genes (IκBα) in subcutaneous adipose tissue, regardless of the quality of dietary fat (SFA from animal fat, 38 E%; MUFA mainly from olive oil, 43 E%; or n3 PUFA, 1.24 g).^25^ These results suggest that pro-inflammatory expression of adipose tissue would be expected among obesity-related diseases. This has been correlated with overproduction of pro-inflammatory adipocytokines. As mentioned earlier, obesity and type 2 diabetes can elicit a pronounced postprandial inflammatory response.^67^

Furthermore, the major characteristic of the Mediterranean diet is a high amount of MUFA (around 20 E%), mainly from olive oil intake.^70^ The Mediterranean dietary pattern has been correlated with reduced cardiovascular morbidity and mortality.^70^^,^^71^ This diet has been encouraged because of its relationship with an improved cardiovascular profile, including its favorable effect on blood pressure, insulin sensitivity, lipid profiles, lipoprotein particles, oxidative stress, carotid atherosclerosis and inflammation.^11^^,^^72^ In a study investigating the effect of consumption of a diet rich in extra-virgin olive oil (MUFA; 21 E%) containing additional Mediterranean components (i.e. fatty fish, unrefined grain products, nuts, legumes and red wine), no effect was found on PBMC inflammatory genes. The same result was obtained from a MUFA intervention (20 E%) consisting of refined olive oil added to a Western-type diet. The Mediterranean diet reduced the plasma concentrations of pro-inflammatory proteins (IL-1β and MIP-1α) after an eight-week intervention, but this change did not significantly differ from interventions consisting of MUFA (20 E%) or SFA (19 E%).^30^ Higher fruit and vegetable consumption has been correlated with lower plasma concentration of CRP and downregulation of pro-inflammatory genes (ICAM-1, IL1-R1, IL-6, TNF-α and NF-κB1) in PBMCs.^10^

In addition to high MUFA content, olive oil contains other minor biologically active components (e.g. polyphenols and carotenoids),^68^ which have been shown to downregulate human genes (IFN-γ, Rho GTPase-activating protein 15 (ARHGAP15) and IL-7R) relating to the inflammatory process.^73^ Thus, essential nutrients such as folate, vitamin C and magnesium, and other bioactive compounds (e.g. flavonoids and carotenoids) that can be found in a Mediterranean dietary pattern, along with olive oil, could be responsible for its anti-inflammatory properties, in addition to the MUFA content. Thus, the factors mentioned may not accurately reveal the role of MUFA diets in relation to the inflammatory response.

Polyunsaturated fatty acids (PUFA) such n-3 and n-6 fatty acids are essential nutrients for health. Recent studies have identified potential benefits from n3 PUFA consumption for a wide range of conditions, including enhancement of the lipid profile^74^ and reduction of coronary heart disease events^75^ and breast cancer risk.^76^ Among severely obese patients (≥ 40 kg/m^2)^ who were scheduled to undergo elective bariatric surgery, n3 PUFA supplementation (3.36 g/day) over an eight-week period showed downregulation of chemokine promoter genes (MCP-1, MIP-1α, HIF1Α and CD40) and a tendency towards reducing IL-6 and increasing anti-inflammatory gene expression of adiponectin in subcutaneous tissue, but not in visceral adipose tissue, in comparison with the control group.^31^ Additionally, dyslipidemic subjects exhibited higher numbers of downregulated pro-inflammatory genes after long-term supplementation with n3 PUFA (2.7 g/day) consisting of fish oil, over a 12-week period, in contrast with subjects with normal lipid levels. Moreover, n3 PUFA showed immune-modulatory and anti-inflammatory capability, through downregulating several pro-inflammatory genes and giving rise to balanced up and downregulation of anti-inflammatory genes, particularly regarding dyslipidemic subjects.^36^ Among elderly individuals, long-term n3 PUFA supplementation via fish oil (26 weeks) showed downregulation of inflammatory pathways in PBMC gene profiles, through 1.8 g/day of EPA/DHA.^29^

Thus, consumption of n3 PUFA seems to improve the inflammatory condition associated with metabolic disorders, in relation to obesity, including insulin resistance and hyperinsulinemia. However, consumption of 1.8 g/day of n3 PUFA via fish oil capsules did not affect inflammatory genes in PBMCs or inflammatory markers (CRP, IL-6 and TNF-α) in insulin-resistant subjects with a mean body mass index of 29.9 kg/m^2^ (standard error of the mean, SEM, 0.9 kg/m^2)^, after eight weeks of supplementation.^34^ In fact, studies have shown divergences regarding the beneficial effects relating to n3 PUFA consumption among individuals with type 2 diabetes mellitus, probably due to the genetic background associated with ethnicity, considering that Asian individuals appear to benefit from supplementation, unlike those of Western origin.^21^^,^^77^^,^^78^ Furthermore, n3 PUFA intake (3.5 E%) of plant origin (2.1 E%) and marine origin (1.4 E%) over a 14-week period did not affect the adipose tissue inflammation in overweight to moderately obese subjects (28-33 kg/m^2)^.^32^ In duodenal tissue, Labonté et al. evaluated the inflammatory response among obese patients with type 2 diabetes following a n3 PUFA intervention (3 g/day) from fish oil for eight weeks. The results failed to demonstrate any significant effect from n3 PUFA supplementation on the gene expression of pro-inflammatory cytokines in duodenal cells.^35^ These authors suggested that the lack of effect was attributable to the low expression of those markers and therefore that they were unlikely to be further modified. However, their study focused on a small number of markers (IL-6, IL-18 and TNF-α) and did not assess any anti-inflammatory markers.

## FINAL CONSIDERATIONS

In summary, the studies reviewed here indicate that MUFA intake and n3 PUFA intake exhibit anti-inflammatory profiles or at least a less pronounced pro-inflammatory response, particularly in comparison with SFA consumption. However, some conflicting results have been described in comparing the inflammatory effects between them. The variability in doses of MUFA (20 E% to 72 E%) and n3 PUFA (0.4 g to 23.7 E%) that were used in interventions may have led to these conflicting results. In addition, the variability in intestinal microbiota among individuals seems to be involved in this postprandial inflammatory response. In this regard, the adaptation of gut microbiota over time may be relevant, especially in comparing acute and long-term effects, but this remains to be determined.

Some other limitations that complicate direct comparisons between the studies deserve further attention. There are differences between the specific populations investigated (i.e. in relation to age, sex, genotype, presence of low-grade inflammation and health status). Methodological factors such as study design, dietary intervention (types of oils, percentage fat and dietary components) and intervention period also differed between the studies reviewed here. Moreover, inflammatory responses were assessed in different tissues (adipose tissue, duodenal tissue, muscle, PBMCs and whole blood), and the inflammatory markers that were screened also differed between the studies. Nevertheless, there is a lack of consensus regarding which biomarker is best for determining inflammation in human nutritional studies.^79^ In this regard, a combination of multiple inflammatory markers appears to be more informative,^79^ although intervention studies have generally focused on a small number of biomarkers instead of several, such as in analyses using microarray methodology. Little emphasis has been placed on anti-inflammatory markers. Thus, regarding the best choice for SFA replacement, limited evidence can support MUFA or PUFA as a better substitute. Identifying the optimal fat profile for inflammatory control may be a promising approach for treating chronic diseases.

## CONCLUSIONS

The evidence indicates that inflammatory gene expression is regulated by the type of fat consumed. In this regard, saturated fatty acid (SFA) consumption has been correlated with a pro-inflammatory response upregulating several genes relating to inflammatory pathways, such as CD16A, MCP-1, MMP-9, IL-1, IL-6, TNFα and p65, in PBMCs and adipose tissue. On the other hand, monounsaturated fatty acid (MUFA) and polyunsaturated fatty acid (PUFA) consumption exhibit an anti-inflammatory profile and a less pronounced pro-inflammatory response, particularly in comparison with SFAs. Thus, partial replacement of SFA with MUFA or PUFA could be a workable nutritional strategy. However, the evidence for indicating the best unsaturated fatty acid for replacing SFAs remains limited. Identifying the optimal fat profile for inflammatory control may be a promising approach for treating chronic diseases. A larger number of studies is necessary in order to elucidate the beneficial inflammatory modulation induced by consumption of these unsaturated fats.
